# Mealiness and Aroma Drive a Non-Linear Preference Curve for ‘Annurca’ PGI Apples in Long-Term Storage

**DOI:** 10.3390/foods14172990

**Published:** 2025-08-27

**Authors:** Giandomenico Corrado, Alessandro Mataffo, Pasquale Scognamiglio, Maurizio Teobaldelli, Boris Basile

**Affiliations:** Department of Agricultural Sciences, University of Naples Federico II, 80055 Portici, Italy; giandomenico.corrado@unina.it (G.C.); alessandro.mataffo@unina.it (A.M.); pasquale.scognamiglio2@unina.it (P.S.); maurizio@teobaldelli.eu (M.T.)

**Keywords:** *Malus domestica*, cold storage, sensory analysis, preference drivers, mealiness

## Abstract

The ‘Annurca’ apple, an EU Protected Geographical Indication product, undergoes a mandatory post-harvest reddening in the ‘melaio’. This traditional practice enhances color and aroma but initiates detrimental textural degradation, creating a paradox where key quality attributes develop in conflict. This study aimed to characterize the sensory evolution of ‘Annurca’ apples during extended cold storage and its impact on consumer preference. A cohort of 551 untrained consumers evaluated the sensory profile at seven time points over a 221-day cold storage period. Multivariate data analyses were employed to identify preference drivers and define consumer segments. Consumer overall liking and market acceptability followed a significant non-linear, U-shaped trajectory, declining from an initial high (89.4% acceptability) to a minimum at day 159 (46.6% acceptability), before partially recovering. This trend inversely correlated with a peak in perceived mealiness, while hardness and crunchiness remained stable. Juiciness and aroma intensity were consistently identified as powerful positive liking drivers, whereas mealiness was the most significant and consistent negative driver. Sweetness’s importance as a preference driver significantly increased over storage time. Cluster analysis on highly rated samples revealed three distinct consumer preference profiles, challenging the traditional notion of a single ideal ‘Annurca’ apple. This study deconstructs the ‘melaio’ paradox, demonstrating that sensory evolution is a dynamic process defined by a trade-off between flavor development and textural decay. The findings provide a data-driven framework for optimizing the commercial strategy for this unique PGI cultivar, suggesting the need to mitigate mealiness and develop targeted marketing strategies for distinct consumer segments.

## 1. Introduction

The ‘Annurca’ apple (*Malus domestica* cv. ‘Annurca’), a cultivar deeply embedded in the agricultural heritage of Southern Italy, holds significant economic and cultural value, underscored by its Protected Geographical Indication (PGI) status as ‘Melannurca Campana PGI’ [1,2]. Its consumer appeal is rooted in a distinct sensory profile, traditionally characterized by a white, firm, and crunchy pulp, a complex sweet aroma, and a pleasantly acidic taste [1]. Beyond its organoleptic qualities, the ‘Annurca’ is noted for a high concentration of bioactive polyphenols, contributing to its nutritional value and antioxidant capacity [3].

The fruit’s characteristically short stem, which makes it prone to premature abscission, necessitates harvesting before full maturity and coloration. Unlike modern cultivars bred for on-tree ripening, this trait is a key part of the Annurca’s unique genetic and cultural identity [2]. Fruits then undergo a unique and mandatory post-harvest reddening process known as the ‘melaio’ stage. This traditional system involves arranging the unripe apples on soft bedding (e.g., straw, wood chips) in open-field structures called ‘melai’. Over a period of a minimum of 10 days, dictated by ambient weather conditions, the fruit is manually turned to ensure uniform exposure to sunlight. This practice is designed to trigger a cascade of biochemical changes, primarily the synthesis of anthocyanins in the peel to achieve the desired deep red coloration. Concurrently, the ‘melaio’ is fundamental for the development of the cultivar’s distinctive flavor and aroma, which is associated with an increase in volatile organic compounds (VOCs) [4,5].

However, this traditional ripening method presents a significant scientific and commercial paradox. While the ‘melaio’ is indispensable for achieving typical visual and aromatic attributes, the same open-field conditions are documented to initiate detrimental textural degradation, linked to a significant loss of flesh firmness and the development of mealiness [6]. The latter is an undesirable sensory attribute characterized by a soft, dry, and disintegrating tissue structure that impacts consumer acceptance and marketability [7,8]. This loss of textural integrity is primarily attributed to enzymatic pectin degradation, which is accelerated during this post-harvest ripening stage [7]. Moreover, the apple’s aromatic profile also evolves in a complex and sometimes conflicting manner. While the ‘melaio’ process is known to increase non-aromatic phenolic esters linked to antioxidant activity in the peel [9], other research indicates that the key volatile esters responsible for the apple’s characteristic aroma remain at low levels following cold storage [5]. These findings underscore the dynamic nature of the apple’s maturation, where different compounds are distinctly affected by the reddening processes.

To extend the commercial window for ‘Annurca’ apples and expand its market reach, producers employ long-term cold storage, a standard practice for most modern cultivars [10]. This strategy, however, introduces questions about how such storage conditions will affect the unique and complex sensory profile developed during the ‘melaio’ stage. During refrigeration, significant physicochemical alterations continue to occur [11]. Desirable flavor and aromatic compounds may degrade while negative textural attributes, such as mealiness, can intensify [7,12]. Mealiness in fruits, and particularly in apples, is a textural defect that arises during ripening or, more often, post-harvest storage [13]. It is characterized by a soft, grainy, and dry mouthfeel resulting from the loss of cell-to-cell adhesion [14]. Instead of rupturing during mastication, cells separate from each other, leading to a perception of dryness and fragmentation in the flesh. For all these reasons, a static assessment of quality is scientifically and commercially insufficient.

A comprehensive characterization of the sensory evolution throughout the entire storage period is a prerequisite for defining reliable shelf-life parameters and ensuring a product that consistently meets both consumer expectations and the demands of the modern retail market. Despite growing interest in post-harvest sensory dynamics, a significant knowledge gap persists. To our knowledge, few studies have comprehensively examined how evolving, and occasionally conflicting sensory attributes influence overall consumer perception over time, mainly because those that exist are often limited to a small number of assessment points [15,16,17,18]. In our specific case, while we understand the immediate effects of the ‘melaio’, it remains unknown how the interplay between desirable flavor development and undesirable textural decay progresses over a commercially relevant storage period. Regrettably, we do not know how these dynamic changes collectively influence consumer liking and acceptability, nor have we identified which sensory attributes become the dominant drivers of preference as the apple ages post-‘melaio’.

Therefore, the present study was conducted to characterize the sensory trajectory of ‘Annurca’ PGI apples following the traditional ‘melaio’ process. Our primary objective was to provide a comprehensive, dynamic understanding of the consumer experience over a six-month storage period. Specifically, this study aimed to (1) quantitatively track the evolution of key sensory attributes, including hardness, crunchiness, mealiness, sweet taste, sour taste, and aroma intensity; (2) model the change in overall consumer liking and define market acceptability thresholds over time; (3) employ multivariate and regression analyses to identify the primary sensory drivers and detractors that govern consumer preference for this cultivar; and (4) investigate whether the relative importance of these sensory drivers changes as a function of storage duration. By systematically mapping the sensory shelf life from the consumer’s perspective, this research seeks to resolve the practical implications of the ‘melaio’ paradox and provide data-driven insights to optimize storage and handling protocols, thereby enhancing the quality consistency and market potential of this unique PGI product.

## 2. Materials and Methods

### 2.1. Experimental Design

A study was conducted to evaluate the effect of post-melaio storage time on the sensory perception of ‘Annurca’ PGI apples. Fruits were harvested at commercial maturity. Its timing was determined by monitoring key physiological indices to ensure they met the mandatory ‘Melannurca Campana PGI’ standards, which, for instance, include a minimum soluble solids content (SSC) of 11.5 °Brix. After harvesting and a 10 days-long reddening process (melaio) (average air temperature of 15.4 °C, with mean minimum and maximum temperatures of 9.9 and 21.5 °C, respectively), a uniform batch of 1500 apples of homogeneous commercial size (caliber: 60–80 mm) was placed in a cold-storage commercial facility equipped with an ethylene extractor and with a temperature and a relative humidity set at 1.0 ± 0.5 °C and 90 ± 5% RH, respectively. The ethylene removal system was operated continuously to maintain concentrations at the low levels typical for commercial apple storage (<0.1 μL L^−1^). Consumer sensory evaluations were conducted at seven distinct time points, at approximately 20–40-day intervals covering six months of cold storage (specifically, 42, 62, 89, 129, 159, 187, and 221 days from harvest).

### 2.2. Consumer Panel

A total of 551 untrained individuals were recruited from the University of Naples Federico II community (Naples, Italy). The panel comprised 206 females, 321 males, and 24 participants who chose not to disclose their gender. All participants were regular consumers of apples, reported no known food allergies, and provided written informed consent prior to their participation in the study.

### 2.3. Sample Preparation

Whole apple samples were allowed to equilibrate to room temperature for one hour. They were then rinsed with fresh tap water, dried, and cut into uniform wedges approximately 30 mm thick. Specifically, after cutting each apple and removing the core, longitudinal wedges were excised from the equatorial region, containing both peel and flesh from the outer cortex to the core line. To prevent identification bias, each apple wedge was placed in a lidded, opaque cup labeled with a random three-digit code. Samples were prepared no more than 10 min before evaluation to minimize enzymatic browning and preserve sensory quality.

### 2.4. Sensory Evaluation Procedure

A monadic sequential evaluation design was employed, where each consumer assessed one coded apple sample per session. Participants were invited to taste each sample and evaluate their overall liking using a 9-point hedonic scale (1 = extremely dislike, 5 = neither like nor dislike, 9 = extremely like). Additionally, they assessed the intensity of specific sensory attributes (i.e., Hardness, Crunchiness, Juiciness, Mealiness, Sweet Taste, Sour Taste, and Aroma Intensity) on a 9-point scale ranging from 1 (very low) to 9 (very high), with 5 representing a medium intensity. To ensure a common understanding of the terms, simple definitions were provided to the panelists in Italian. Hardness was defined as ‘the force needed to bite into the apple wedge’; Crunchiness as ‘the amount of sound produced during chewing’; Juiciness as ‘the amount of liquid released in the mouth’; and Mealiness was explained using the specific Italian term *farinosità*, which was described as ‘the degree to which the apple pulp feels soft and breaks down into small, dry, grainy particles’. Sweet Taste and Sour Taste were defined as the basic taste intensities, and Aroma Intensity was described as ‘the overall intensity of the “apple” flavor and fragrance perceived while chewing’.

Unrefrigerated mineral water and unsalted crackers were provided for palate cleansing, to be used before the test (and during the evaluation if needed). Ratings were collected using a structured paper scoresheet provided to each participant, who also had the opportunity to write a short comment.

### 2.5. Statistical Analysis

All statistical analyses were performed using R v4.3.3 running in RStudio (Rstudio, Boston, MA, USA). Data manipulation and visualization were conducted primarily with the tidyverse package suite. Inferential tests were carried out using the stats package, emmeans for post hoc analysis and factoextra for cluster analysis visualization. A significance level (α) of 0.05 was used for all statistical tests.

#### 2.5.1. Data Preprocessing

Prior to analysis, any consumer response with missing values for the primary outcome, ‘Overall Liking’, was removed from the dataset. The date of each sensory test was converted into two distinct variables: (1) a categorical factor (Test Date) to serve as the independent variable in ANOVA models, and (2) a continuous numeric variable (Storage Time) calculating the days elapsed since harvest for use in regression models. A binary dependent variable, ‘Acceptability’, was derived from the ‘Overall Liking’ score, where scores of ≥6 on the 9-point hedonic scale were coded as acceptable (1) and scores of <6 as not acceptable (0) [19,20].

#### 2.5.2. Analysis of Storage Time Effects

A one-way ANOVA was conducted for each sensory attribute to evaluate the effect of storage time. Post hoc pairwise comparisons were performed using Tukey’s Honestly Significant Difference (HSD) test. The effect of storage time on the binary ‘Acceptability’ outcome was assessed using a binomial logistic regression model. Contingency tables were constructed to summarize the frequency distribution of categorical outcomes across groups. A Pearson’s chi-squared test of independence was employed to evaluate whether there were statistically significant differences in the proportions of ‘Acceptability’ among the seven distinct storage time points.

#### 2.5.3. Multivariate and Predictive Analyses

Principal Component Analysis (PCA) was used to explore the interrelationships between the sensory attributes and to visualize the underlying structure of the sensory data. The data were centered and scaled prior to analysis. A multiple linear regression model was constructed to identify the key sensory drivers of ‘Overall Liking’. To allow for direct comparison of the effect sizes; all sensory attribute predictors were standardized before being entered into the model. A Random Forest (RF) regression model was developed to assess the predictive relationship between sensory attribute ratings and consumer overall liking scores. This non-linear, machine-learning-based approach was selected to capture complex interactions and assess variable importance using permutation importance within the sensory data. The dataset was randomly partitioned into a training set (80% of samples) and a testing set (20%) to evaluate the model’s generalizability. The RF model was constructed with 500 trees (ntree = 500). The mtry hyperparameter, which defines the number of predictors randomly sampled at each split, was explicitly set to 3, a value often chosen for regression models with a moderate number of predictors to balance bias and variance. Missing values were handled by omitting incomplete cases (na.action = na.omit). Model training and internal validation employed the Out-of-Bag (OOB) cross-validation method, providing an unbiased estimate of explained variance (R^2^). Model performance was additionally quantitatively assessed using the root mean squared error (RMSE) on the independent testing set, reflecting the average deviation between predicted and observed overall liking scores. Variable importance measures derived from the model facilitated the identification of key sensory attributes influencing consumer preferences. All modeling was conducted in R (4.3.3) using the randomForest library.

#### 2.5.4. Dynamic Driver and Preference Profile Analyses

To test whether the importance of key attributes (Sweetness, Mealiness) changed over time, linear models including an interaction term between the attribute and continuous storage time were analyzed. Finally, to identify distinct sensory profiles among the most highly rated samples (Overall Liking ≥ 7), a k-means cluster analysis was performed. The optimal number of clusters was determined using the ‘elbow method’ (within-cluster sum of squares). The final cluster centroids were profiled to describe the characteristics of each preferred apple type.

## 3. Results

### 3.1. Consumer Overall Liking

**Figure 1:**
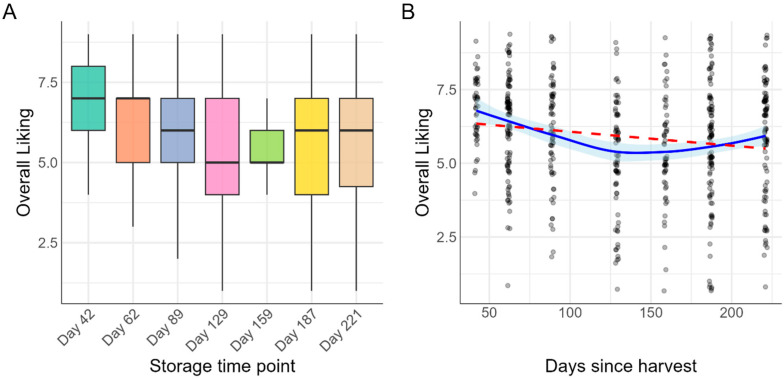
Change in consumer overall liking of ‘Annurca’ apples during post-melaio storage: (**A**) Boxplots showing the distribution of overall liking scores at seven discrete time points. The thick line represents the median score. (**B**) Scatterplot of all individual consumer ratings against storage time (days since harvest). The blue line represents a flexible LOESS regression model with a 95% confidence interval (shaded region), highlighting the non-linear trend in consumer preference.

The overall liking of ‘Annurca’ apples exhibited a significant non-linear trend over the storage period (Figure 1A). Initially, following the melaio process, the apples received high-liking scores. However, scores progressively declined, reaching their lowest point after approximately 159 days of storage. Following this minimum, consumer liking scores began to recover in the subsequent evaluation periods. A flexible LOESS regression model fit to the data confirmed this U-shaped pattern, demonstrating that a simple linear model was inadequate to describe the change in consumer preference over time (Figure 1B).

### 3.2. Effect of Storage Time on Sensory Attributes

Storage time had a significant effect on Overall Liking (F(6, 544) = 6.00, *p* < 0.001). Post hoc analysis with Tukey’s adjustment was performed to identify specific differences between time points (Table 1). The highest liking scores were recorded at the start of the storage period (Day 42: M = 6.83). These scores were significantly higher than those at Day 129 (M = 5.25, *p* < 0.001) and Day 159 (M = 5.45, *p* = 0.002), which represented the lowest points in consumer acceptance. Notably, by the final time point (Day 221: M = 5.89), liking scores had recovered to a level that was not statistically different from the initial scores at Day 42 (*p* = 0.055). This result confirms a significant U-shaped trend in consumer preference over the storage duration.

In contrast to Overall Liking, the one-way ANOVA indicated that there was no statistically significant effect of storage time on perceived Hardness (F(6, 535) = 0.71, *p* = 0.641). Similarly, there was no significant effect of storage time on perceived Juiciness (F(6, 533) = 0.44, *p* = 0.854). Perceived Crunchiness also did not show a significant change over the storage period (F(6, 532) = 1.46, *p* = 0.191).

A significant effect of storage time was also found for Mealiness (F(6, 528) = 4.33, *p* < 0.001). The pattern followed an inverted U-shape, mirroring the trend for Overall Liking. Perceived Mealiness peaked during the winter months, with the highest score recorded on Day 159 (M = 5.71). Subsequently, Mealiness scores significantly decreased, reaching their lowest point at the final evaluation on Day 221 (M = 4.35). Post hoc analysis confirmed that Mealiness at Day 221 was significantly lower than at Day 159, Day 187, and Day 129 (*p* < 0.05) (Table 1).

A significant effect of storage time was also observed for perceived Sweetness (F(6, 542) = 3.22, *p* = 0.004). In contrast to the U-shaped trend of liking, Sweetness showed a general decline over the storage period. The highest Sweetness ratings were recorded at the beginning of the study on Day 42 (M = 6.28) and Day 62 (M = 5.92). By the final evaluation on Day 221, the perceived Sweetness had dropped to its lowest point (M = 5.04), which was significantly lower than the scores from the first two time.points (*p* < 0.05) (Table 1). The perceived Sourness was also significantly affected by storage time (F(6, 538) = 2.20, *p* = 0.041). For most of the evaluation period, the perceived Sourness remained relatively stable. However, there was a significant increase at the final time point (Day 221), where the mean score (M = 4.03) was significantly higher than the lowest recorded score at Day 187 (M = 3.28; *p* < 0.05) (Table 1). Finally, there was no significant effect of storage time on perceived Aroma Intensity (F(6, 535) = 1.46, *p* = 0.190).

To translate these liking scores into a practical measure of market viability, consumer acceptability was also analyzed (Figure 1B). An apple was defined as ‘acceptable’ if it received a liking score of 6 or higher. A logistic regression confirmed that the proportion of acceptable apples changed significantly over time (χ^2^(6) = 30.6, *p* < 0.001). This analysis quantifies the market risk associated with the mid-storage quality dip, showing that consumer acceptability fell from nearly 90% at Day 42 to a low of approximately 47% at Day 159, before recovering to over 70% by the end of the study.

### 3.3. Modeling Consumer Acceptability over Time

**Figure 2:**
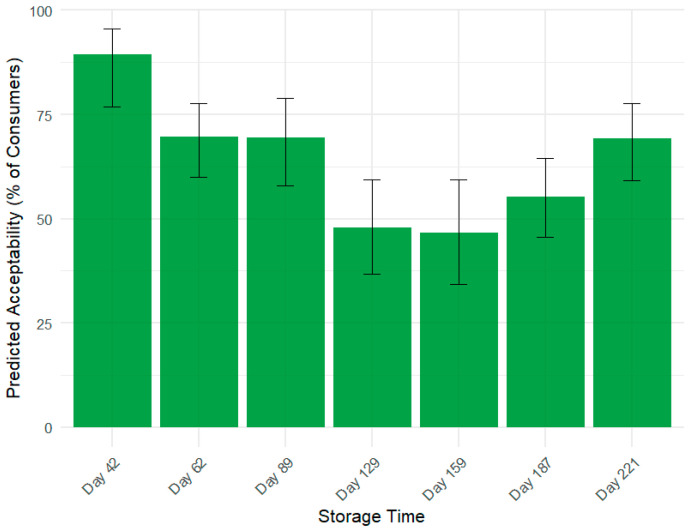
Predicted consumer acceptability of ‘Annurca’ apples over the storage period. Acceptability is defined as the percentage of consumers rating the apple with an ‘Overall Liking’ score of 6 or higher on a 9-point scale. Bars represent the predicted mean percentage derived from a binomial logistic regression model, and error bars show the 95% confidence intervals.

While the average liking score (Figure 1A) indicates the central tendency of consumer opinion, it can obscure crucial information about market risk. To address this, we conducted an acceptability analysis to determine the percentage of the consumer panel that found the product commercially viable, defined as a liking score of 6 or higher. This ‘headcount’ of satisfied consumers provides a more direct measure of market potential than the average rating alone. A binomial logistic regression model was used to robustly estimate the proportion of ‘accepters’ at each storage time point. The model confirmed a highly significant effect of storage time on Acceptability (χ^2^(6) = 30.6, *p* < 0.001). As visualized in Figure 2, the results reveal a dramatic U-shaped trajectory for the apple’s market potential. Initially, the apples showed high commercial promise, with nearly 90% of consumers finding them acceptable (Day 42). However, this proportion plummeted to a low of 47% by Day 159, representing a point of significant commercial risk where fewer than half of consumers would be satisfied. Crucially, in line with the trend in Overall Liking, Acceptability rebounded during the final two months of storage, recovering to 72% by Day 221. This analysis moves beyond a simple average to quantify the dynamic shifts in consumer consensus, demonstrating how the product’s viability in the market falls and subsequently recovers over its shelf life.

### 3.4. Principal Component Analysis of Sensory Attributes

**Figure 3:**
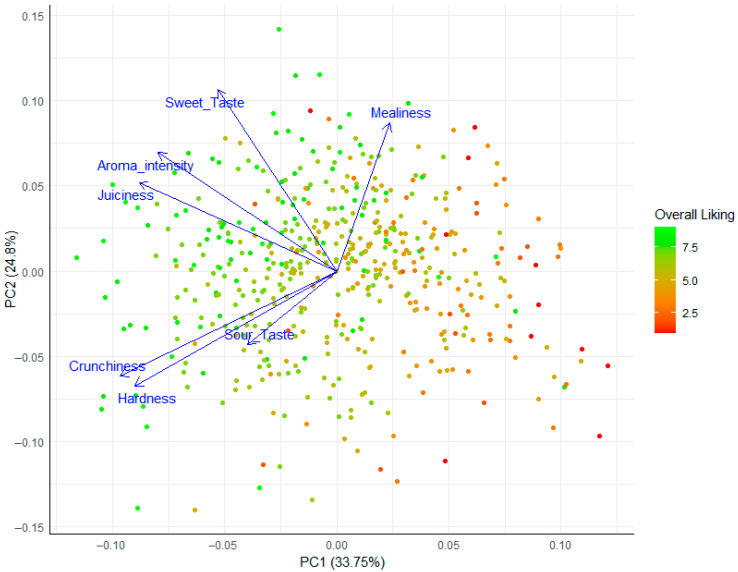
Principal Component Analysis (PCA) biplot of the seven sensory attributes. Individual consumer ratings are plotted as points and colored by the ‘Overall Liking’ score, from low (red) to high (green). The blue vectors represent the original sensory attributes, showing their direction and contribution to the first two principal components. The percentage of variance explained by each component is shown on the axis labels.

To explore the underlying structure of the sensory data and visualize the relationships between attributes, a Principal Component Analysis (PCA) was performed. The first two principal components (PC1 and PC2) collectively explained 58.6% of the total variance in the sensory data (33.8% and 24.8%, respectively). This moderate level of explained variance suggests that the selected sensory attributes are not highly redundant but rather capture distinct aspects of the consumer’s perceptual experience. The biplot of the results is presented in Figure 3. The first Principal Component (PC1) clearly separated the attributes based on quality perception. Hardness, Crunchiness, Juiciness, Sweet Taste, and Aroma Intensity loaded negatively on PC1, while Mealiness loaded positively. This component can thus be interpreted as a primary ‘Sensory Quality’ dimension, contrasting desirable textural and flavor attributes with the negative attribute of Mealiness. The second component (PC2) primarily distinguished between texture (Hardness, Crunchiness) and flavor/aroma attributes.

Critically, when consumer scores were colored by their ‘Overall Liking’ rating, a clear pattern emerged. High liking scores (green points) were predominantly located on the left side of the plot, strongly associated with the vectors for Hardness, Crunchiness, and Juiciness. Conversely, low liking scores (red points) were concentrated on the right-hand side of the plot, aligned with the Mealiness vector. This visualization generated the hypothesis that consumer liking for the ‘Annurca’ apple is positively driven by its textural characteristics (firmness, crunchiness, and juiciness) and is strongly negatively driven by perceptions of mealiness. While PCA was used for visualizing data structure, we applied multiple linear regression and Random Forest models to identify key drivers of consumer preference and test hypotheses.

### 3.5. Identifying the Key Drivers of Consumer Liking

**Figure 4:**
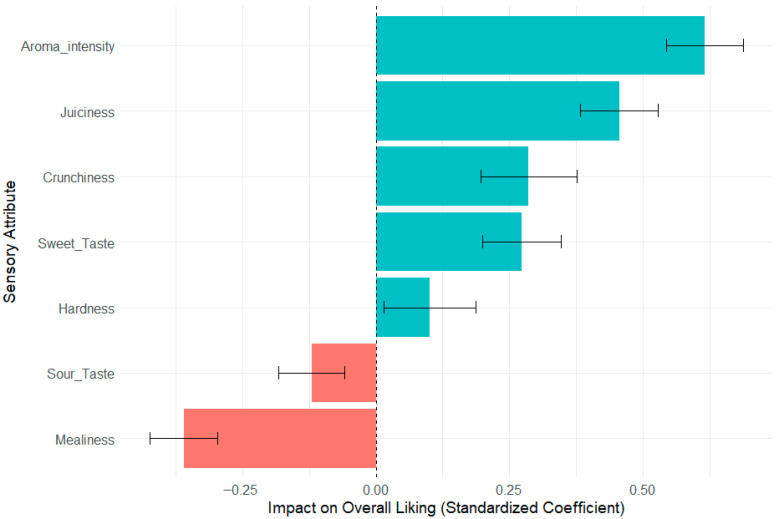
Key drivers of ‘Overall Liking’ for ‘Annurca’ apples. The plot displays the standardized regression coefficients (β) for each sensory attribute from a multiple linear regression model. The length of the bar indicates the strength of the impact on the liking score, while the error bars represent the standard error of the coefficient. Positive coefficients (blue) indicate positive drivers of liking, while negative coefficients (red) indicate negative drivers.

To quantify the relative importance of each sensory attribute in predicting consumer preference, a multiple linear regression model was built with ‘Overall Liking’ as the outcome variable. All sensory attributes were included as scaled predictor variables to allow for direct comparison of their impact (Figure 4 and Supplementary Table S1).

The model was highly significant (F(7, 521) = 65.9, *p* < 0.001, R^2^ = 0.47) and identified a clear hierarchy of sensory drivers (Figure 3). Perceived Aroma Intensity was the strongest positive driver of Overall Liking (β = 0.62, *p* < 0.001), followed by Juiciness (β = 0.46, *p* < 0.001), Crunchiness (β = 0.29, *p* = 0.002), and Sweet Taste (β = 0.27, *p* < 0.001). Conversely, Mealiness was the most significant negative driver (β = −0.36, *p* < 0.001). In this comprehensive model, Hardness and Sour Taste were not found to be significant predictors of Overall Liking. These results confirm that consumer preference for ‘Annurca’ apples is primarily driven by high aroma intensity and a juicy, crunchy texture, while being strongly penalized by the presence of mealiness.

### 3.6. Predictive Modeling and Driver Confirmation with Random Forest

To complement the explanatory linear models and to account for any potential non-linear relationships or complex interactions between attributes, a Random Forest regression model was employed. This machine learning approach provides a robust, non-parametric method for assessing the predictive power of the sensory profile and identifying key drivers of consumer preference.

#### 3.6.1. Model Performance and Predictive Accuracy

The Random Forest model, constructed with 500 trees, demonstrated a meaningful ability to predict consumer liking scores based on the sensory attribute ratings. The model explained 37.7% of the variance in ‘Overall Liking’ based on the OOB internal cross-validation estimate. This R-squared value indicates a moderate but significant predictive relationship, which is substantial for complex and inherently variable human sensory data.

**Figure 5:**
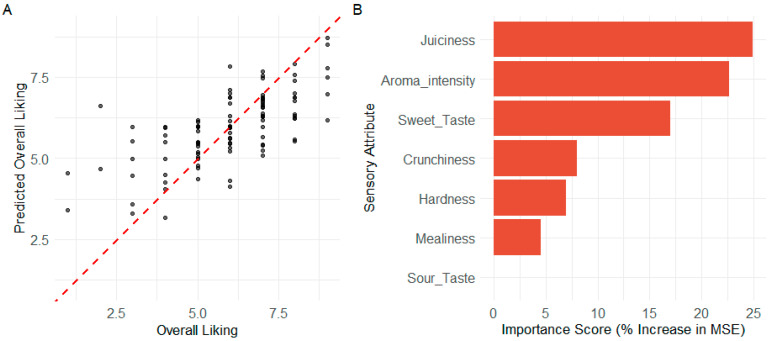
Random Forest model performance and variable importance: (**A**) Predicted versus actual ‘Overall Liking’ scores on the unseen test data. The dashed red line represents perfect prediction. (**B**) Variable importance plot ranking the sensory attributes based on the percentage increase in mean squared error (%IncMSE) when the variable’s values are randomly shuffled. Higher values indicate greater importance to the model’s predictive accuracy.

When applied to the unseen test set, the model’s predictive accuracy was quantified with an RMSE of 1.32. This metric signifies that, on average, the model’s predictions deviate from the actual consumer liking scores by approximately 1.3 points on the 9-point hedonic scale. A visual representation of the model’s predictive performance (Figure 5A) shows a clear positive correlation between predicted and actual scores, though with the expected degree of variance reflective of the RMSE value.

#### 3.6.2. Variable Importance and Driver Hierarchy

A key output of the Random Forest is the quantification of variable importance, which measures how much the model’s accuracy degrades when the information from a specific attribute is removed. This analysis provides a robust, model-agnostic ranking of the sensory drivers. The importance scores, calculated as the percentage increase in mean squared error (%IncMSE), are presented in Figure 5B. The analysis reveals a distinct hierarchy of drivers. Juiciness (%IncMSE ≈ 25) and Aroma Intensity (%IncMSE ≈ 22) were unambiguously identified as the two most critical predictors of consumer liking. The model relied heavily on information from these two attributes to make its predictions. A second tier of important positive drivers included Sweet Taste (%IncMSE ≈ 17) and Crunchiness (%IncMSE ≈ 8).

Notably, this non-linear model assigned lower importance to Mealiness (%IncMSE ≈ 5) compared with the linear regression. This suggests that while Mealiness is a significant linear detractor, its overall predictive impact may be less dominant than that of top-tier positive drivers like Juiciness and Aroma when complex interactions are considered. In strong agreement with all previous analyses, Sour Taste was found to have negligible importance, confirming it was not a primary driver of Overall Liking for this product. Collectively, these findings from the Random Forest analysis corroborate and enrich the conclusions from the linear models, providing powerful, converging evidence for the key sensory attributes that define the consumer experience of ‘Annurca’ apples.

### 3.7. Gender-Based Differences in Sensory Perception and Liking

An exploratory analysis was conducted to evaluate whether gender influenced sensory perception, overall liking, or the relationship between sensory attributes and consumer preferences for ‘Annurca’ apples. Independent t-tests were used to compare mean ratings between female and male participants across Overall Liking and the seven sensory attributes. In addition, interaction models were constructed to test whether gender moderated the relationship between the individual sensory attributes and Overall Liking.

The results revealed no significant difference in Overall Liking scores between female and male consumers (*p* = 0.895). Among the sensory attributes, only Juiciness showed a statistically significant difference: female consumers rated apples as slightly more juicy (mean = 5.61) than male consumers (mean = 5.29; t(373.7) = 1.98, *p* = 0.048). All other sensory attributes (e.g., Hardness, Crunchiness, Sweetness, Aroma Intensity) showed no significant gender-based differences (*p* > 0.1).

Furthermore, interaction analyses indicated that the effect of sensory attributes on Overall Liking did not differ between genders. All interaction terms were non-significant, with the lowest *p*-value observed for Crunchiness (*p* = 0.100). This suggests that male and female consumers rely on the same sensory drivers when evaluating apple liking.

Given the consistency of findings across attributes and models, and the marginal nature of the one significant result, these analyses support the conclusion that gender does not meaningfully influence sensory perception or preference in this context (Supplementary Table S2).

### 3.8. Dynamic Drivers: How the Importance of Attributes Changes with Storage

To investigate whether the drivers of consumer liking evolve during long-term cold storage, a final analysis explored the interaction between the key sensory attributes and storage time. This approach tests the hypothesis that the importance of an attribute to a consumer might change depending on whether the apple is perceived as ‘fresh’ (early storage) or ‘old’ (late storage). Based on the primary findings of this study, Sweetness and Mealiness were selected as the most relevant attributes for this dynamic analysis. Specifically, Sweetness was chosen because its perceived intensity changed linearly over time, while Mealiness was selected as it was identified as the primary driver of the U-shaped liking curve.

For easier interpretation of the results and more effective display, the continuous storage time was grouped into three distinct periods: ‘Short Storage’ (Days 42–62), ‘Medium Storage’ (Days 89–129), and ‘Long Storage’ (Days 159–221).

**Figure 6:**
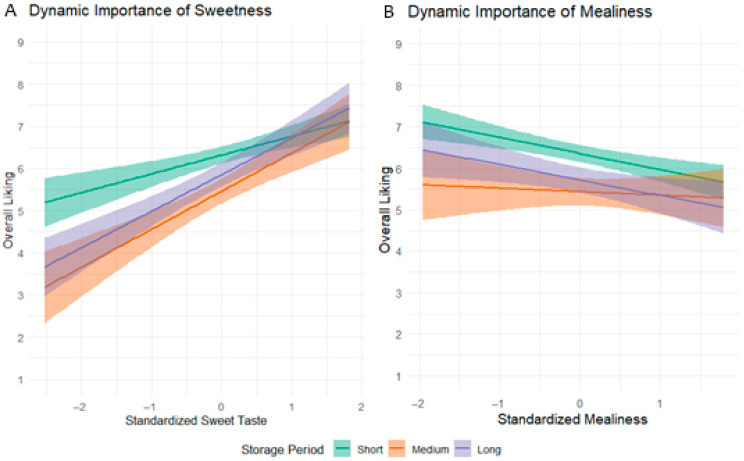
The dynamic effect of Sweetness and Mealiness on Overall Liking across different storage periods. The plots show the interaction between a sensory attribute (x-axis) and storage time (colored lines) in predicting ‘Overall Liking’ (y-axis). The steeper the slope, the stronger the attribute’s impact on liking. (**A**) The importance of Sweetness as a driver of liking significantly increases (interaction *p* = 0.034). (**B**) The negative impact of Mealiness on liking remains consistently strong across all storage periods (interaction *p* = 0.868).

A significant interaction was found between Sweet Taste and storage time (β = 0.16, *p* = 0.034). As visualized in Figure 6A, the positive impact of Sweetness on Overall Liking becomes significantly stronger as storage progresses. For apples in the ‘Short Storage’ period, the relationship is moderately positive. However, for apples in the ‘Medium’ and ‘Long’ storage periods, the slope of the relationship steepens considerably. This indicates that while sweetness is always desirable, it becomes a more critical determinant of liking in apples stored for longer periods.

In contrast, no significant interaction was found between Mealiness and storage time (*p* = 0.868). The visualization in Figure 6B confirms this, showing three nearly parallel regression lines. This result demonstrates that the negative impact of Mealiness on consumer liking is consistently strong and does not significantly change throughout the entire 221-day storage period. Mealiness is an equally powerful detractor regardless of the length of the cold storage.

### 3.9. Identifying Preferred Sensory Profiles via Cluster Analysis

To determine if distinct sensory profiles exist among the most highly rated apples, a k-means cluster analysis was performed on all samples that received an ‘Overall Liking’ score of 7 or higher. An elbow plot analysis suggested an optimal solution of three clusters (Supplementary Figure S1), indicating three distinct preference segments among consumers. The defining characteristics of these three profiles are summarized in Table 2.

The two largest preference segments, ‘Profile 1’ (36.0%) and ‘Profile 2’ (37.4%), were both driven by high Sweetness. Profile 1, the ‘Sweet & Soft’ profile, was characterized by high Sweetness combined with low Sourness, but also low Hardness and Crunchiness. Profile 2, the ‘Intensely Flavored & Juicy’ profile, scored highest on Aroma Intensity, Juiciness, and Sweetness. Notably, this largest segment also exhibited the highest level of perceived Mealiness, suggesting that for these consumers, a powerful flavor and juiciness experience can override a significant textural defect (Table 1).

In contrast, the third segment, ‘Profile 3’ (26.6%), represented a ‘Crisp, Firm, & Tart’ preference. This profile was defined by its superior textural qualities, scoring highest on Hardness and Crunchiness and lowest on Mealiness. The trade-off for this textural excellence was a flavor profile with the lowest Sweetness and highest Sourness. These results indicate that there is not one singular ideal ‘Annurca’ apple, but rather several distinct and desirable sensory profiles that appeal to different consumer segments.

## 4. Discussion

This study investigates the sensory evolution of the ‘Annurca’ PGI apple during the critical six-month storage period that follows the traditional *melaio* process. By mapping consumer perception, this research addresses the consequences of the well-known ‘melaio’ paradox (flavor is enhanced at the cost of textural integrity) to provide a dynamic understanding of how this trade-off unfolds over a commercially relevant timeframe. The results provide a comprehensive, dynamic characterization of consumer experience, revealing the complex interplay of sensory attributes that govern liking and acceptability. The central finding is the significant non-linear, U-shaped trajectory for both consumer liking and market acceptability, a pattern directly linked to the evolving textural and flavor profile of the apple post-melaio.

The initial high liking scores immediately following the melaio process validate the traditional knowledge behind this practice. However, fruit quality is a dynamic attribute, and maintaining consumer satisfaction during extended storage remains a major industry challenge [21]. It is therefore not surprising that this optimal condition is transient. The subsequent significant decline in consumer liking, reaching the lowest point at approximately 129–159 days of storage, coincides with the peak in perceived Mealiness. This finding empirically confirms the detrimental textural degradation initiated during the open-field reddening stage. Interestingly, our analysis revealed no significant change in perceived Hardness or Crunchiness over the entire storage period. This suggests that the negative textural attribute of Mealiness is a distinct perceptual construct from firmness and is the primary driver of textural rejection in ‘Annurca’ apples. This finding is consistent with other consumer research, which identifies mealiness as a relevant attribute that generally causes most consumers to reject apples [21,22,23]. This also indirectly confirms that Mealiness is best scored by a sensory analysis [24,25].

Despite the recognized irreversibility of mealiness, interestingly, our data revealed a reversal in consumer liking. Previous studies indicated that controlled atmosphere storage can sustain sensory favorability over time, but this was mainly linked to the preservation of textural properties [26]. In our case, the rebound was concurrent with a significant decrease in perceived Mealiness, suggesting a partial resolution of this key textural defect. However, this recovery was not a simple return to the initial state. Crucially, the rebound in liking occurred alongside a fundamental shift in the flavor profile (i.e., a significant decrease in perceived Sweetness and an increase in Sourness) while other positive attributes like Juiciness and Aroma Intensity remained stable. This complex dynamic strongly suggests that the recovery is not driven by a physical reversal of texture, but rather by a perceptual modulation, considering also that mealiness is typically considered irreversible. We propose that the emergence of this distinct, tarter flavor profile alters the overall sensory experience, making the remaining textural defects more acceptable to consumers. This creates a ‘late-storage’ ‘Annurca’ apple that is different from its ‘fresh’ post-melaio counterpart yet presents a distinct sensory profile that nevertheless achieves comparable levels of consumer acceptance.

To move to a quantitative understanding, we identified which sensory attributes were most influential in driving the overall U-shaped liking curve. The convergence of Principal Component Analysis (PCA), multiple linear regression, and Random Forest models provides robust evidence for a clear hierarchy of sensory drivers [27]. The PCA visually partitioned the sensory space along a primary axis (PC1) that contrasted desirable attributes (Juiciness, Crunchiness, Aroma) with Mealiness, effectively representing a ‘Sensory Quality’ dimension. High liking scores were unequivocally clustered with positive textural and aromatic vectors, while low liking scores were dominated by the Mealiness vector.

Building directly on this visual evidence, the regression models were then used to formally quantify these relationships. The results identified Aroma Intensity and Juiciness as the most powerful positive predictors of overall liking, while Mealiness was the most significant detractor. The Random Forest model, which accounts for non-linearities and interactions, corroborated the importance of Juiciness and Aroma Intensity. The comparatively lower importance assigned to Mealiness by the Random Forest model suggests that while Mealiness is a potent linear detractor, its negative impact may be partially mitigated or ‘overridden’ in the presence of exceptionally high levels of positive drivers. This explanation was supported by the cluster analysis findings. This complex interplay helps reconcile the ‘melaio’ paradox: the process simultaneously boosts a primary driver (Aroma) while introducing a primary detractor (Mealiness).

A key contribution of this study is the demonstration that the importance of sensory drivers is not static but evolves with storage time [21]. The significant interaction between Sweet Taste and storage time reveals that as the apple ages, sweetness becomes an increasingly critical determinant of liking [28]. In fresh, post-melaio apples, other complex flavors and textural cues may dominate preference; however, as the apple transitions into late storage, consumers appear to rely more heavily on the fundamental perception of sweetness as an indicator of quality [28]. Conversely, Mealiness was found to be an unwavering and powerful detractor throughout the entire storage period. Its negative impact on liking did not diminish over time, establishing it as the principal and most consistent sensory defect to be managed. This has profound practical implications: while flavor profiles may be tailored to different consumer segments, the mitigation of mealiness must be a universal and primary goal for extending the commercial life of the ‘Annurca’ apple. This reinforces the importance of exploring interventions like 1-MCP treatment to specifically target pectin degradation pathways [6], to extend the commercial life of the ‘Annurca’ apple and prevent the negative consumer experiences that lead to brand or cultivar switching [29].

While the regression models identified universal preference drivers, the k-means cluster analysis challenged the notion of a single ‘ideal’ ‘Annurca’ apple. Instead, our findings identify three distinct and viable sensory profiles that appeal to different consumer segments [30]. The largest segment, ‘Intensely Flavored & Juicy’ (37.4%), is arguably the most insightful. These consumers grant the highest liking scores to apples that excel in aroma, juiciness, and sweetness, and are remarkably tolerant of the highest levels of mealiness. For this group, a powerful flavor and juice experience can successfully override significant textural defects. This profile represents consumers who embrace the outcome of the ‘melaio’ trade-off.

In contrast, the ‘Crisp, Firm, & Tart’ segment (26.5%) represents a more traditionalist preference, prioritizing superior texture (high hardness/crunchiness, low mealiness) at the expense of lower sweetness and aroma. The ‘Sweet & Soft’ profile (36.0%) represents a third pathway, prioritizing sweetness while being less demanding on texture and aroma. These findings provide a strategic roadmap for the PGI consortium. Rather than a one-size-fits-all quality standard, they can develop targeted strategies, potentially sorting fruit post-storage to match these distinct consumer preference profiles, thereby maximizing market value and consumer satisfaction. This is consistent with studies that have identified predominant consumer segments with differing preferences, such as those who prefer ‘sweet and hard’ apples versus those who prefer ‘juicy and acidic’ ones [31,32,33]. Lastly, the absence of significant gender-based differences in Overall Liking or the drivers of preference simplifies the marketing model, suggesting that segmentation strategies should focus on sensory preferences rather than demographics [34,35].

Ultimately, our findings provide an actionable roadmap for the PGI consortium. Technically, the primary goal should be the mitigation of mealiness through targeted interventions, such as optimizing 1-MCP treatment timing post-*melaio* [36]. Commercially, our results suggest moving towards a dynamic marketing strategy. Apples in early storage (the first ~3 months) should be marketed to consumers who prioritize a classic and expected crisp–firm texture. Apples in late storage (after 5–6 months), which develop a tarter, more intense flavor profile, could be targeted to the apple industry or to a different consumer segment that values flavor complexity over perfect texture [37,38].

## 5. Conclusions

This study successfully deconstructed the consumer experience of ‘Annurca’ PGI apples, providing critical insights to navigate the post-melaio sensory evolution and optimize commercial strategy. A key finding is that consumer liking of ‘Annurca’ apples follows a distinct U-shaped curve over six months of cold storage, a trajectory driven by an initial development of desirable aroma, a mid-storage decline due to a peak in mealiness, and a late-storage recovery as mealiness subsides, albeit with an altered flavor profile. This dynamic reflects a fundamental clash between the most powerful positive drivers of liking (Juiciness and Aroma Intensity) and the most significant and consistent negative driver, Mealiness. Furthermore, the study revealed that the importance of these drivers is dynamic; as storage progresses, the importance of sweetness as a quality indicator intensifies, while the detrimental impact of mealiness remains constantly high. Finally, our research reveals that the market for ‘Annurca’ apples consists of at least three distinct consumer segments with different sensory preferences, some of whom are willing to tolerate textural defects in exchange for distinctive flavors. This finding challenges the traditional belief of a single ‘perfect’ ‘Annurca’ apple, showing instead that multiple profiles are desirable.

Our work provides a data-driven framework for the ‘Annurca’ PGI consortium to move beyond traditional, static quality assessments. By understanding how sensory attributes evolve and how they differentially impact distinct consumer segments, producers can optimize storage and handling protocols, define more sophisticated, dynamic shelf-life parameters, and implement targeted marketing strategies that enhance the consistency, consumer acceptance, and market potential of this unique and culturally significant apple cultivar.

## Figures and Tables

**Table 1:** Effect of storage time on consumer sensory perception and acceptability of apples. Data for sensory attributes are presented as mean scores ± standard deviation. All attributes were rated on a 9-point hedonic scale (1 = dislike extremely, 9 = like extremely). Acceptability is shown as percentage of consumers who found the sample acceptable. Within each column, mean values are compared using Tukey’s HSD test. Means that do not share a common letter are significantly different (*p* < 0.05). Letters are only shown for attributes where the overall effect of storage time was statistically significant in the main ANOVA. N represents the number of consumers per storage time condition.

Storage Time (Days)	N	Sensory Attribute								
		Hardness	Crunchiness	Juiciness	Mealiness	Sweet Taste	Sour Taste	Aroma Intensity	Overall Liking	Acceptability
42	47	4.28 ± 1.64	4.78 ± 1.75	5.66 ± 1.67	5.34 ± 1.98 ab	6.28 ± 1.50 b	3.40 ± 1.61 ab	5.60 ± 1.39	6.83 ± 1.11 d	89.4% b
62	102	4.21 ± 1.70	4.56 ± 1.89	5.48 ± 1.93	5.03 ± 2.26 ab	5.92 ± 1.88 b	3.43 ± 1.75 ab	5.70 ± 1.84	6.35 ± 1.64 cd	69.6% ab
89	72	4.28 ± 1.60	4.54 ± 2.12	5.51 ± 1.64	4.83 ± 2.35 ab	5.71 ± 1.95 ab	3.81 ± 1.83 ab	5.48 ± 1.67	6.14 ± 1.66 bcd	69.4% ab
129	73	4.10 ± 1.93	4.15 ± 2.09	5.35 ± 1.40	5.48 ± 2.12 b	5.37 ± 1.84 ab	3.43 ± 1.69 ab	5.07 ± 1.72	5.25 ± 1.90 a	47.9% a
159	58	3.82 ± 1.79	4.16 ± 1.76	5.39 ± 1.34	5.71 ± 1.71 b	5.62 ± 1.62 ab	3.81 ± 1.54 ab	5.12 ± 1.68	5.45 ± 1.74 ab	46.6% a
187	105	4.04 ± 1.97	4.02 ± 2.04	5.27 ± 1.81	5.67 ± 2.11 b	5.48 ± 1.92 ab	3.28 ± 1.87 a	5.29 ± 1.82	5.65 ± 2.08 abc	55.2% a
221	94	4.37 ± 1.87	4.45 ± 1.78	5.29 ± 1.89	4.35 ± 1.96 a	5.04 ± 2.03 a	4.03 ± 1.73 b	5.22 ± 1.85	5.89 ± 1.91 abcd	69.1% ab

**Table 2:** Sensory characteristics of the three preferred ‘Annurca’ apple profiles identified by k-means cluster analysis. The analysis was performed on all consumer ratings where ‘Overall Liking’ was ≥7 on a 9-point scale. Values represent the mean sensory score (cluster centroid) for each attribute within a profile. The ‘Size (%)’ column indicates the percentage of highly liked ratings that belong to each profile.

Profile	Size (%)	Hardness	Crunchiness	Juiciness	Mealiness	Sweet Taste	Sour Taste	Aroma Intensity
Profile 1	36.0%	3.03	3.33	5.80	4.96	6.30	2.76	5.72
Profile 2	37.4%	5.06	5.81	7.06	5.80	7.63	3.70	6.99
Profile 3	26.6%	6.39	6.75	5.95	3.00	4.98	4.38	5.95

## Data Availability

The anonymized consumer sensory dataset generated during this study is available from the corresponding author (Boris Basile, boris.basile@unina.it) upon reasonable request.

## Supplementary Materials

The following supporting information can be downloaded at https://www.mdpi.com/article/10.3390/foods14172990/s1, Figure S1: Determination of the optimal number of clusters (k) using the Elbow Method. The plot displays the total within-cluster sum of squares (WSS) as a function of the number of clusters tested (from k = 1 to 10). The “elbow” of the curve, which represents the point of diminishing returns where adding more clusters provides little additional explanatory power, is observed at k = 3; Table S1: Summary of linear regression model assessing the influence of scaled sensory attributes on Overall Liking. The table presents estimated regression coefficients (Estimate), standard errors (Std. Error), t-values, and *p*-values for each predictor variable. Significance levels are indicated as follows: *** *p* < 0.001; ** *p* < 0.01; ns: not significant; Table S2: Summary of Statistical Tests for Gender Differences in Sensory Perception and Overall Liking. The table presents results from independent t-tests comparing mean scores for Overall Liking and individual sensory attributes between female (Mean F) and male (Mean M) participants. It also includes the t-statistics, p-values, and significance for the interaction terms from regression models, evaluating whether gender moderates the relationship between sensory attributes and Overall Liking (Liking Driver Interaction). NA indicates ‘not applicable’ for interaction rows as they do not represent simple means. Significance levels are: * *p* < 0.05; n.s.: not significant at *p* ≥ 0.05.
