# Supplementary material for: Mealiness and Aroma Drive a Non-Linear Preference Curve for ‘Annurca’ PGI Apples in Long-Term Storage
Source: Foods. 2025 Aug 27;14(17):2990. doi: 10.3390/foods14172990 (PMC12428182; doi:10.3390/foods14172990)
Supplement: Supplementary file 1 [file foods-14-02990-s001.zip › Supplementary Material.pdf]

**Supplementary Table S1.** Summary of linear regression model assessing the influence of scaled sensory attributes on Overall Liking. The table presents estimated regression coefficients (Estimate), standard errors (Std. Error), t-values, and p-values for each predictor variable. Significance levels are indicated as follows: ***p < 0.001; **p < 0.01; ns: not significant

| Predictor | Estimate | Std. Error | t value | Pr(>\|t\|) | Significance |
|---|---|---|---|---|---|
| (Intercept) | 5.93976 | 0.05830 | 101.891 | < 2e-16 | *** |
| Hardness (scaled) | 0.10082 | 0.08610 | 1.171 | 0.242156 | n.s |
| Crunchiness (scaled) | 0.28637 | 0.08977 | 3.190 | 0.001513 | ** |
| Juiciness (scaled) | 0.45565 | 0.07267 | 6.270 | 7.91e-10 | *** |
| Mealiness (scaled) | -0.35981 | 0.06365 | -5.652 | 2.69e-08 | *** |
| Sweet Taste (scaled) | 0.27348 | 0.07381 | 3.705 | 0.000235 | *** |
| Sour Taste (scaled) | -0.12032 | 0.06231 | -1.931 | 0.054054 | n.s. |
| Aroma intensity (scaled) | 0.61534 | 0.07212 | 8.533 | < 2e-16 | *** |

**Supplementary Table S2.** Summary of Statistical Tests for Gender Differences in Sensory Perception and Overall Liking. The table presents results from independent t-tests comparing mean scores for Overall Liking and individual sensory attributes between female (Mean F) and male (Mean M) participants. It also includes the t-statistics, p-values, and significance for the interaction terms from regression models, evaluating whether gender moderates the relationship between sensory attributes and Overall Liking (Liking Driver Interaction). NA indicates 'not applicable' for interaction rows as they do not represent simple means. Significance levels are: *p < 0.05; n.s.: not significant at p ≥ 0.05.

| Analysis | Attribute | Mean F | Mean M | Statistic | Value | P Value | Significance |
|---|---|---|---|---|---|---|---|
| Overall Liking | Overall Liking | 5.945 | 5.969 | t(343.2) | -0.13 | 0.895 | n.s. |
| Sensory Perception | Hardness | 4.230 | 4.117 | t(347.8) | 0.64 | 0.520 | n.s. |
| Sensory Perception | Crunchiness | 4.574 | 4.320 | t(358.2) | 1.35 | 0.178 | n.s. |
| Sensory Perception | Juiciness | 5.612 | 5.292 | t(373.7) | 1.98 | 0.048 | * |
| Sensory Perception | Mealiness | 4.989 | 5.302 | t(358.5) | -1.52 | 0.130 | n.s. |
| Sensory Perception | Sweet Taste | 5.557 | 5.746 | t(364) | -1.07 | 0.286 | n.s. |
| Sensory Perception | Sour Taste | 3.519 | 3.622 | t(355) | -0.61 | 0.543 | n.s. |
| Sensory Perception | Aroma Intensity | 5.372 | 5.471 | t(382.7) | -0.61 | 0.544 | n.s. |
| Liking Driver Interaction | Hardness | NA | NA | t-value (interaction) | -0.66 | 0.512 | n.s. |
| Liking Driver Interaction | Crunchiness | NA | NA | t-value (interaction) | -1.65 | 0.100 | n.s. |
| Liking Driver Interaction | Juiciness | NA | NA | t-value (interaction) | -0.59 | 0.556 | n.s. |

| | | | | | | | |
|---|---|---|---|---|---|---|---|
| Liking Driver Interaction | Mealiness | NA | NA | t-value (interaction) | 0.78 | 0.438 | n.s. |
| Liking Driver Interaction | Sweet Taste | NA | NA | t-value (interaction) | 0.53 | 0.599 | n.s. |
| Liking Driver Interaction | Sour Taste | NA | NA | t-value (interaction) | 0.02 | 0.984 | n.s. |
| Liking Driver Interaction | Aroma Intensity | NA | NA | t-value (interaction) | -1.63 | 0.103 | n.s. |

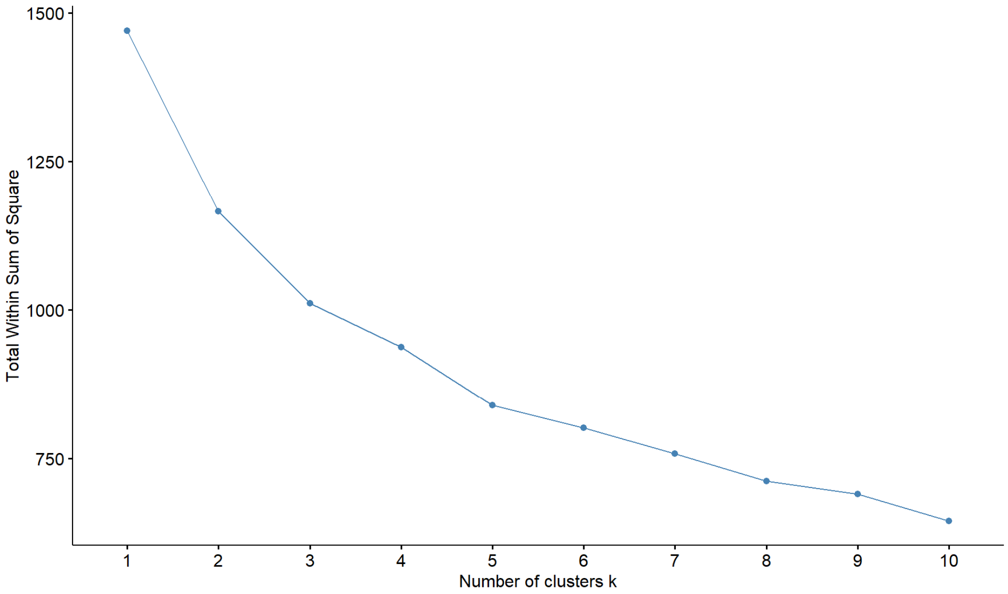


**Supplementary Figure S1.** Determination of the optimal number of clusters (k) using the Elbow Method. The plot displays the total within-cluster sum of squares (WSS) as a function of the number of clusters tested (from k=1 to 10). The "elbow" of the curve, which represents the point of diminishing returns where adding more clusters provides little additional explanatory power, is observed at k=3.
